# Combined Cardiac Arrhythmias Leading to Electrical Chaos Developed in the Convalescent Phase of SARS-CoV-2 Infection: A Case Report and Literature Review

**DOI:** 10.3390/jcm14176053

**Published:** 2025-08-27

**Authors:** Emilie Han, Ena Hasimbegovic, Robert Schönbauer, Dietrich Beitzke, Mariann Gyöngyösi

**Affiliations:** 1 Division of Cardiology, Department of Internal Medicine II, Medical University of Vienna, A-1090 Vienna, Austria; 2 Department of Biomedical Imaging and Image-Guided Therapy, Medical University of Vienna, A-1090 Vienna, Austria

**Keywords:** case report, long COVID, complex cardiac arrhythmia

## Abstract

**Background**: Acute SARS-CoV-2 infection may induce cardiac arrhythmias associated with viral myocarditis, which typically disappear in the convalescent phase after healing of the myocardial inflammation. **Methods**: We report the case of a 37-year-old woman with a childhood history of atrial septal defect repair and stable normofrequent atrial rhythm, who presented two months post-COVID-19 with palpitations and dizziness. Diagnostic evaluation included cardiac magnetic resonance imaging (CMR), 24 h Holter electrocardiogram (ECG) monitoring, and laboratory assessments over a 3-year period. **Results**: CMR suggested subacute myocarditis, and Holter ECG revealed multiple discernible complex cardiac arrhythmias including atrial bradycardia, intermittent junctional rhythm (JR), atrial fibrillation (AF), and non-sustained ventricular tachycardia. Laboratory results showed a moderate but transient increase in lactate dehydrogenase, persistently mildly elevated N-terminal pro–B-type natriuretic peptide (NT-proBNP), and immunoglobulin A (IgA), with all other cardiac, inflammatory, immunologic, and organ function parameters remaining normal. In spite of chaotic cardiac rhythm with alternating JR, AF, and atrial normofrequent rhythm with frequent blocked supraventricular beats and increasing atrioventricular conduction time, no therapeutic intervention was necessary during follow-up, and a conservative treatment approach was agreed with the patient. Two years post-COVID-19 infection, the patient returned to a normofrequent atrial rhythm with a markedly prolonged PQ time (500 ms) and a different P wave morphology compared to pre-COVID, without other rhythm disturbances. **Conclusions**: This case demonstrates a rare pattern of post-viral arrhythmias first emerging in the convalescent phase and resolving spontaneously after two years. It underscores the need for long-term rhythm surveillance following COVID-19, even in patients with prior structural heart disease and a stable baseline rhythm.

## 1. Introduction

Acute COVID-19 manifests in multiple organs involving the cardiovascular system with varying degrees of severity. The incidence of acute viral myocarditis is 150–4000 cases/100,000 individuals infected with SARS-CoV-2 [1]. Fewer reports delve into heart rhythm issues, in spite of a 4–10% incidence of atrial fibrillation/flutter during acute infection [2]. Emerging evidence shows that late-onset arrhythmias can develop even after mild SARS-CoV-2 infection, though more data exists for acute COVID-19 patients with severe symptoms [3]. Myocardial inflammation caused by viral infection may lead to prolonged low-grade inflammation or dysfunctional immune response, which could contribute to inflammatory cardiac channelopathies and conduction abnormalities [4]. Furthermore, it is known that in patients who recovered from acute cardiac injury, remaining myocardial fibrosis can be detected by cardiac magnetic resonance imaging (CMR) [5]. Such residual fibrotic remodeling may disrupt normal cell-to-cell communication and electrical coupling, reducing conduction efficiency and predisposing the patient to palpitations and arrhythmias [6]. This falls in line with one proposed hypothesis for the pathophysiology of long COVID based on autonomic nervous system dysfunction and decreased heart-rate variability [6,7]. The interplay among structural, inflammatory, and autonomic factors may trigger a spectrum of rhythm disturbances, including an increased susceptibility to both brady- and tachyarrhythmias, observed in COVID-19 convalescent patients. Nevertheless, in the setting of long COVID, detailed reports of cases with complex cardiac arrhythmias, such as the one presented here, are lacking.

## 2. Case Presentation

**Figure 1:**
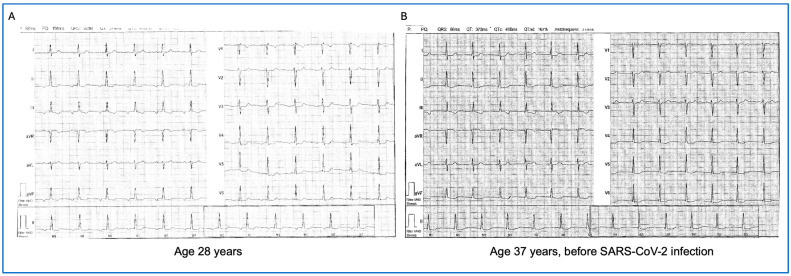
Pre-COVID infection electrocardiograms from doctor’s office visits: Normofrequent basal atrial rhythm with negative P waves in the inferior leads (**A**) and asymptomatic junctional escape rhythm 9 years later (**B**).

A 37-year-old female patient presented at our clinic with palpitations that had first occurred 2 months after her acute SARS-CoV-2 infection. Detailed clinical history revealed previous surgical closure of an atrial septal defect at 3 years old, and she had not exhibited any clinical cardiac symptoms since then. Sinus arrest with an asymptomatic rhythmic normofrequent atrial rhythm developed after cardiac surgery and can be observed in the electrocardiogram (ECG) from her yearly doctor’s office visit (Figure 1A). This rhythm changed to an asymptomatic junctional escape rhythm recorded before infection with SARS-CoV-2 (Figure 1B).

**Figure 2:**
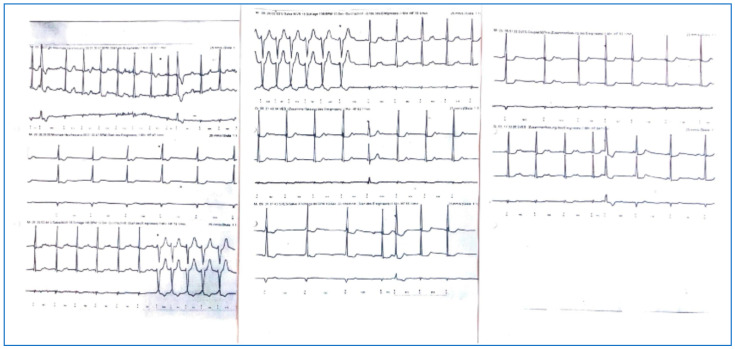
Atrial tachycardia with intermittent junctional rhythm with intermittent episodes of sinus rhythm with distinct 1st-degree AV block, isolated ventricular extrasystoles, and monomorphic non-sustained ventricular tachycardia; 24 h ECG, 2 months after COVID-19 infection.

At the time of infection, the patient had not been vaccinated and suffered from flu-like symptoms and fatigue and recovered at home. Two months later, due to newly developed recurrent palpitations, she underwent ambulatory 24 h ECG monitoring, which demonstrated atrial bradycardia with a prolonged PQ time, alternating with supraventricular tachycardia and intermittent junctional escape rhythm, with a minimal heart rate of 47 beats per minute (bpm) and non-sustained ventricular tachycardia of 16 ventricular ectopic beats with a maximal rate of 106 bpm lasting 10 s (Figure 2). Unfortunately, at that time, the 24 h ECG findings were misinterpreted, and no further action was taken in the primary care setting.

Due to ongoing complaints, 3 months after SARS-CoV-2 infection, the patient underwent cardiac magnetic resonance imaging (CMR). Discrete edema formation and accentuated early arterial enhancement in the mid-inferolateral section of the ventricular myocardium were observed, while the left ventricular ejection fraction was mildly reduced, which was interpreted as subacute viral myocarditis. Because laboratory analyses revealed no abnormalities, no further treatment was indicated.

**Figure 3:**
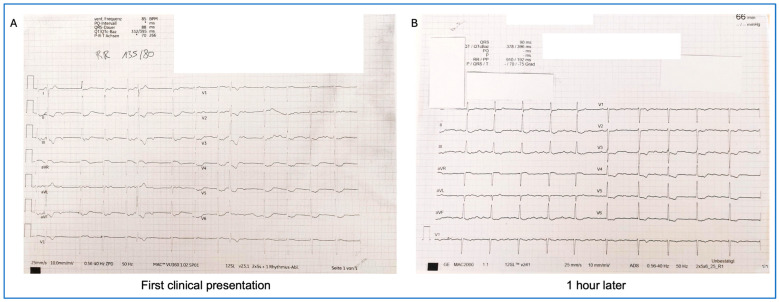
ECG at first clinical presentation. Chaotic cardiac rhythm with accelerated junctional rhythm with retrograde P waves at a heart rate of 85 bpm, recurrent premature ventricular complexes and post-extrasystole block of the junctional rhythm to the ventricle, simulating 3rd-degree AV block (**A**). ECG 1 h later. De novo paroxysmal atrial flutter with 240 ms cycle length and changing conduction to the ventricle (**B**).

Two months later, due to persistent symptoms, the patient was referred to our outpatient long COVID cardiac care unit and included in the POSTCOV Registry (EC: 1008/2021). The patient, who was a non-athlete of normal constitution (164 cm, 62 kg, BMI 23 kg/m^2^), reported palpitations of varying duration and dizziness, mainly presenting at rest. Physical examination showed no abnormal findings except discernible arrhythmia during auscultation. The patient was a sales assistant and non-smoker, had no known allergies, no home medication, and no known autoimmune disease. On the initial ECG at our outpatient clinic, chaotic cardiac rhythm was observed with accelerated junctional rhythm and retrograde P waves at a heart rate of 85 bpm. Additionally, isolated premature ventricular beats and post-extrasystole blocked atrial beats, simulating 3rd-degree AV block could be observed (Figure 3A). Transthoracic echocardiography was performed immediately, but the left ventricular functional parameters and dimensions were within normal range (left ventricle size: 47 mm; intraventricular septum, 4-chamber view: 7 mm).

On the second ECG, which was performed less than 1 h after the initial recording, we detected de novo atrial flutter with an average ventricular rate of 90 bpm (Figure 3B). Full spectrum of laboratory investigations were performed, including quantitative hemogram (complete blood count and white blood cell differential); coagulation status (PT/INR, aPTT, fibrinogen, D-dimer, von-Willebrand-factor antigen, and ADAMTS 13 activity); electrolytes (Na, K, Cl, Ca, and Mg); cardiac markers; inflammatory biomarkers (C-reactive protein, lactate dehydrogenase (LDH), immunoglobulins, interleukin 6, procalcitonin, and ferritin, tryptase); liver and kidney function parameters (ALAT, ASAT, GGT, AP, creatinine, blood urea nitrogen, uric acid, and albumin); metabolic markers (glucose, HbA1c, triglycerides, cholesterol, and amylase); and endocrinological markers (TSH, histamine, and homocysteine). Complete blood count, coagulation status, electrolytes, and liver and kidney function parameters were in the normal range. N-terminal pro-B-type natriuretic peptide (NT-proBNP) (370 pg/mL, ref: 0–125) and LDH (344 U/L, ref: <250) levels were elevated with normal troponin T (<4 ng/L, ref: 0–14) and creatine kinase (CK) (91 U/L, ref: <170) levels. No clinical or laboratory signs of active infection were detected. C-reactive protein (0.09 mg/dL, ref: <0.5) was not elevated. A serological investigation for infectious pathogens (herpes simplex, herpes zoster virus, Epstein–Barr virus, parvovirus B19, cytomegalovirus, and SARS-CoV-2) excluded acute viral infection. Serum immunoglobulin A (IgA) level was mildly elevated (461 mg/dL, ref: 70–400). All other measured laboratory markers returned values within the normal range. All laboratory analyses were performed at the Clinical Institute of Laboratory Medicine (KILM) of the Medical University of Vienna, where information for analytical methods and analyzer information can be found in the parameter list (found online at https://akhwien.at/default.aspx?pid=3985, accessed on 14 August 2025). The patient was admitted overnight for additional ECG monitoring, and oral anticoagulation was initiated with edoxaban (per os 60 mg once daily).

The next day, transesophageal echocardiography (TEE) detected no thrombi in the atria or atrial appendages, no residual shunting across the atrial septal wall, but a thick left ventricular false tendon. Electrical cardioversion on the same day was performed, but without success. In the next days, serial ECGs were performed, which showed accelerated junctional rhythm switching to atrial fibrillation (AF) and flutter with a mean ventricular rate of 80 bpm. Considering the normal immunologic and cardiac laboratory parameters, and to investigate the chronotropic response to exercise, a stress test was performed on a cycle ergometer, which showed AF with chronotropic competence. Recurrent polymorphic ventricular ectopic beats occurred with ventricular salvoes (max. 3 beats), but the patient remained oligosymptomatic throughout the exercise test. The reason for termination was peripheral muscle fatigue after the patient reached the minimum test duration of 12 min with a maximum 125 W (calculated maximum: 128 W). Due to suspected binodal dysfunction, no antiarrhythmic drugs were initiated for the home medication therapy. The patient was frequently followed up in our outpatient care, but she remained oligosymptomatic, and the ECG displayed permanent AF.

Two months after the initial patient presentation at our long COVID clinic, repeated CMR showed normalization of the left ventricular ejection fraction (64%) and no signs of cardiac edema or ventricular late enhancement.

**Figure 4:**
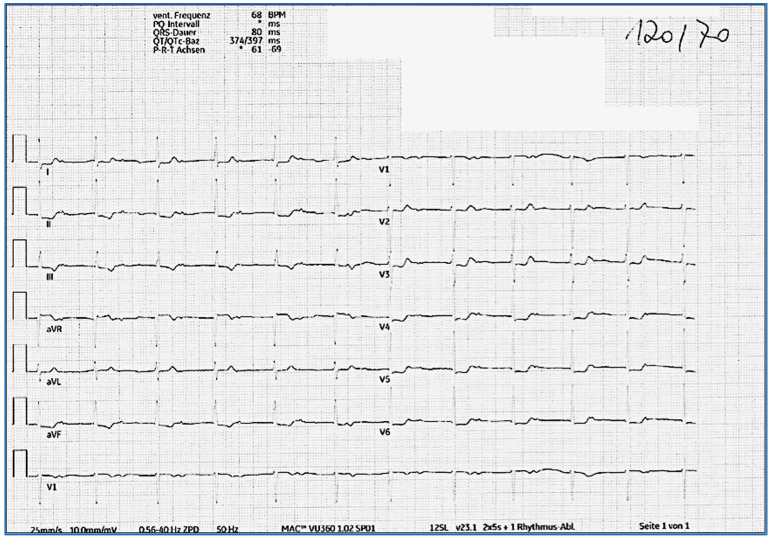
ECG 3 years after COVID-19 infection. Normofrequent atrial rhythm with antegrade conduction with a 1st-degree AV block (PQ > 500 ms).

At the 1-year follow-up visit, a repeated ECG and 24 h ECG showed frequent switching between intermittent AF and accelerated JR with a ventricular frequency between 50 and 100 bpm, though she reported no symptoms. During the frequent clinical follow-up investigations, she reported no symptoms, and she started her regular training similar to pre-COVID disease. Several discussions were performed with the patient regarding the necessity of an electrophysiological investigation. However, the patient was subjectively symptom-free, and a repeated Holter ECG did not show ventricular tachycardia or bradycardia with any pauses, and the cardiac MR findings were normalized. Thus, it was agreed to not perform an invasive investigation, but to keep up with frequent clinical follow-ups. At 2 years post-COVID infection, since no AF or flutter episodes were detected in the 24 h ECGs and the patient had a CHA2DS2–VASc score of 1, the decision was made to stop anticoagulation. In the most recent ECG, 3 years after the initial presentation, the patient presented with normofrequent atrial rhythm with antegrade conduction with a first-degree atrioventricular block (PQ > 500 ms; Figure 4). Notably, the morphology of the atrial P wave changed compared with that of the pre-COVID morphology, indicating a shift in the site of atrial pacemaker activity.

Three years after a mild COVID-19 infection and after developing protracted combined chaotic heart rhythm disturbances for 1 year, the patient currently has no clinical symptoms and feels like her general health has improved again; she goes jogging for a hobby (~5 km/week, same as pre-COVID). The patient comes to regular follow-ups at the cardiac outpatient care unit.

A full timeline and overview of the clinical case can be found in Table 1.

## 3. Discussion

Rhythm changes observed on the ECG (e.g., supraventricular tachycardias, malignant ventricular dysrhythmias, bradycardia, and AV block) have been reported during the acute phase of SARS-CoV-2-infected patients, as well as in post-COVID individuals, though the reported frequency is comparably less in the latter group [8,9,10,11,12,13]. However, chaotic cardiac rhythm with occasionally combined life-threatening atrial and ventricular rhythm disturbances and conduction abnormalities developing several months after the acute viral infection have not yet been reported in post-COVID patients.

After surgical closure of the ASD in childhood, the patient developed a stable junctional escape rhythm without bradycardia or pause, with no symptoms; therefore, further invasive investigations and pacemaker implantation were avoided. The first symptomatic malignant cardiac arrhythmias were associated with the post-acute COVID illness, resulting in diverse supraventricular and ventricular rhythm disturbances, finally leading to atrial rhythm with antegrade conduction delay (1st-degree AV block) and relocation of the atrial myocyte with pacemaker function during the 3-year-long COVID phase.

Elevation of circulating cardiac and inflammatory parameters support the diagnosis of acute myocarditis. Patients with acute COVID-19-induced myocarditis often have elevated troponin T, CK, and NT-proBNP and a range of inflammatory markers including C-reactive protein, interleukin-6, D-dimer, ferritin, fibrinogen, facultatively also von-Willebrand-factor antigen/activity, and various positive autoimmune parameters. In most cases, these laboratory abnormalities resolve during the convalescent phase [2]. In contrast, all laboratory values of our patient remained in the normal range, apart from the transient elevation of LDH and the persistently mild elevation of NT-proBNP and serum IgA. The elevation of NT-proBNP might be the consequence of rhythm disturbances. An elevated level of IgA is characteristic for several diseases, such as IgA nephropathy, systemic vasculitis, monoclonal gammopathies, and chronic inflammatory diseases or genetic disorders, such as Wiskott–Aldrich syndrome. A temporary elevated IgA level has been described in newborns with congenital heart diseases [14], but a persistently elevated or decreased level of IgA has also been measured in diverse congenital cardiovascular aberrations [15]. The persistently elevated circulating IgA level of our patient even 3 years after the SARS-CoV-2 infection could instead be associated with her congenital heart disease and not with the healed post-COVID-19 infection. The elevated level of IgA persisted after the disappearance of the COVID-19-induced arrhythmias (AF or non-sustained VT), and the patient had no vasculitis or any other inflammatory disorders, which makes a relation between IgA and cardiac arrhythmias unlikely. Since the patient had not received any COVID vaccination prior to the appearance of rhythm disturbances, the possibility of vaccine-induced myocarditis and associated rhythm disturbances was excluded.

Autonomic dysfunctions, including orthostatic intolerance, which were described in long COVID patients from previous studies [16,17], may amplify arrhythmogenic risk. A potential pathophysiological mechanism includes atrial or ventricular myocyte injury mediated by inflammatory cytokines through the course of myocarditis and viral invasion [3]. SARS-CoV-2 has shown tropism possibly facilitated by cardiomyocytes and pericytes expressing ACE2 and thus able to directly cause cardiac injury [18,19]. Furthermore, the SARS-CoV-2 spike protein can cause fusing of human cardiomyocytes and the formation of syncytia, which may be a potential pathomechanism for arrhythmic diseases developing post-COVID [20]. Concurrently, inflammation-induced atrial remodeling can underlie subsequent AF and binodal dysfunction contributing to mixed rhythm disturbances [21], as seen in this case report. While CMR data directly after COVID cases is sparse, extrapolations from viral myocarditis demonstrate that myocardial fibrosis and gap junction disruption (e.g., connexin remodeling) can cause delayed conduction abnormalities and arrhythmogenesis [22]. Lastly, atrial cardiomyopathy could be a potential cause for the structural and electrical remodeling leading to these complex arrhythmias as observed in this case [23,24]. Whether or not a previous infection with SARS-CoV-2 prompts the development of atrial cardiomyopathy is still unclear, as current literature on this association is limited.

## 4. Conclusions

Late-onset post-COVID cardiac rhythm disturbances may reflect persistent conduction system remodeling in the heart and autonomic imbalance following subclinical myocarditis. In our case, binodal dysfunction, polymorphic ventricular ectopy, and non-sustained ventricular tachycardia emerged months after apparent recovery. Clinical monitoring with serial ECGs and Holter ECGs should be performed for post-COVID patients with palpitations or cardiac complaints.

## Figures and Tables

**Table 1:** Summary of the findings.

Time	Event or Finding	Consequence
Pre-COVID infection	Electrocardiogram (ECG): normofrequent junctional escape rhythm after surgical closure of the atrial septal defect	Asymptomatic, adequate chronotropic response to stress, no bradycardia or syncope: Patient’s choice to avoid electrophysiology investigation and pacemaker implantation. Regular follow-ups instead.
Baseline	COVID-19 infection	-
2 months later	24 h ECG at primary care: misinterpreted combined arrhythmia	-
At 4 months	Cardiac magnetic resonance imaging (CMR) suggested subacute myocarditis with edema and slightly reduced left ventricular ejection fraction Laboratory investigation at primary care: cardiac enzymes and inflammatory parameters in normal range (numeric values not reported)	-
At 6 months (first clinical presentation at the outpatient long COVID cardiac care unit)	Laboratory testing: Anti-nuclear antibodies and other autoantibodies (against cardiolipin IgG, cardiolipin IgM, beta-2 glycoprotein IgG, beta-2 glycoprotein IgM) all negative; cardiac enzymes negative (troponin T < 4 ng/L, ref: 0–14) mildly elevated LDH (344 U/L, ref: <250), NT-proBNP (370 pg/mL, ref: 0–125), and serum immunoglobulin-A (461 mg/dL, ref: 70–400) ECG: chaotic cardiac rhythm with accelerated junctional rhythm with retrograde P waves, 85 bpm One hour later, de novo atrial flutter	Transthoracic echocardiography (TTE): normal left ventricular function Overnight admission with 24 h ECG monitoring Initiation of 60 mg edoxaban once daily (CHADS-VASc-Score 1)
Next day	Transesophageal echocardiography (TEE): no intracardiac thrombi detected	Electric cardioversion without success, continued anticoagulation and frequent follow-ups in heart rhythm outpatient care
At 8 months	Repeated CMR: recovered left ventricular ejection fraction (LVEF 64%), no signs of cardiac edema or ventricular late enhancement Resting ECG: alternating junctional escape rhythm and atrial fibrillation Exercise testing: alternating junctional escape rhythm and atrial fibrillation, polymorphic ventricular extrasystoles, ventricular couplets and triplets, but no complaints during testing Laboratory testing: mildly elevated NT-proBNP (236 pg/mL, ref: 0–125) and serum immunoglobulin-A (454 mg/dL, ref: 70–400); all other laboratory testing normal	Continuation of 60 mg edoxaban once daily Considered electrophysiology investigation, but the patient was asymptomatic Close follow-up in heart rhythm outpatient care
At 11 months	Improving symptoms and no complaints, no dizziness	No indication for ablation of atrial fibrillation due to oligosymptomatic and ventricular normofrequency and possible risk of necessary pacemaker implantation after ablation
1-year follow-up	ECG: alternating atrial fibrillation with accelerated junctional rhythm Laboratory testing: mildly elevated NT-proBNP (453 pg/mL, ref: 0–125) and serum immunoglobulin-A (409 mg/dL, ref: 70–400); all other laboratory testing normal	Continue anticoagulation, frequent clinical and heart rhythm clinic follow-ups
2-year follow-up	ECG: normofrequent atrial rhythm with prolonged PQ time (>500 ms) with antegrade conduction. P wave morphology is different compared to that of pre-COVID ECG 24 h ECG: no atrial fibrillation episodes	Decision to stop anticoagulation; continue regular yearly clinical, laboratory, and rhythmic follow-ups
3-year follow-up	ECG: normofrequent atrial rhythm with prolonged PQ time (>500 ms) with antegrade conduction Laboratory testing: mildly elevated NT-proBNP (328 pg/mL, ref: 0–125) and serum immunoglobulin-A (469 mg/dL, ref: 70–400)	Continue with regular clinical, laboratory, and rhythmic follow-ups

## Data Availability

All data is included in this paper.

## Abbreviations

The following abbreviations are used in this manuscript:

AF	Atrial fibrillation
ASD	Atrial septal defect
AV	Atrioventricular
Bpm	Beats per minute
CK	Creatine kinase
CMR	Cardiac magnetic resonance imaging
ECG	Electrocardiogram
IgA	Immunoglobulin A
JR	Junctional rhythm
LDH	Lactate dehydrogenase
LVEF	Left ventricular ejection fraction
NT-proBNP	N-terminal pro-b-type natriuretic peptide
VT	Ventricular tachycardia

## References

[1] Fairweather D., Beetler D.J., Di Florio D.N., Musigk N., Heidecker B., Cooper L.T. (2023). COVID-19, Myocarditis and Pericarditis. Circ. Res..

[2] Saha S.A., Russo A.M., Chung M.K., Deering T.F., Lakkireddy D., Gopinathannair R. (2022). COVID-19 and Cardiac Arrhythmias: A Contemporary Review. Curr. Treat. Options Cardiovasc. Med..

[3] Gopinathannair R., Olshansky B., Chung M.K., Gordon S., Joglar J.A., Marcus G.M., Mar P.L., Russo A.M., Srivatsa U.N., Wan E.Y. (2024). Cardiac Arrhythmias and Autonomic Dysfunction Associated With COVID-19: A Scientific Statement from the American Heart Association. Circulation.

[4] Tsampasian V., Bäck M., Bernardi M., Cavarretta E., Dębski M., Gati S., Hansen D., Kränkel N., Koskinas K.C., Niebauer J. (2025). Cardiovascular Disease as Part of Long COVID: A Systematic Review. Eur. J. Prev. Cardiol..

[5] Brendel J.M., Klingel K., Gräni C., Blankstein R., Kübler J., Hagen F., Gawaz M., Nikolaou K., Krumm P., Greulich S. (2024). Multiparametric Cardiac Magnetic Resonance Imaging to Discriminate Endomyocardial Biopsy-Proven Chronic Myocarditis from Healed Myocarditis. JACC Cardiovasc. Imaging.

[6] Marques K.C., Quaresma J.A.S., Falcão L.F.M. (2023). Cardiovascular Autonomic Dysfunction in “Long COVID”: Pathophysiology, Heart Rate Variability, and Inflammatory Markers. Front. Cardiovasc. Med..

[7] Qin M., Lee K., Yoo S.-J. (2025). The Impact of Long COVID on Heart Rate Variability: A Cross-Sectional Study. BMC Infect. Dis..

[8] Parotto M., Gyöngyösi M., Howe K., Myatra S.N., Ranzani O., Shankar-Hari M., Herridge M.S. (2023). Post-Acute Sequelae of COVID-19: Understanding and Addressing the Burden of Multisystem Manifestations. Lancet Respir. Med..

[9] Soriano J.B., Murthy S., Marshall J.C., Relan P., Diaz J.V. (2022). A Clinical Case Definition of Post-COVID-19 Condition by a Delphi Consensus. Lancet Infect. Dis..

[10] Long B., Brady W.J., Bridwell R.E., Ramzy M., Montrief T., Singh M., Gottlieb M. (2021). Electrocardiographic Manifestations of COVID-19. Am. J. Emerg. Med..

[11] Cimino G., Pascariello G., Bernardi N., Calvi E., Arabia G., Salghetti F., Bontempi L., Vizzardi E., Metra M., Curnis A. (2020). Sinus Node Dysfunction in a Young Patient With COVID-19. JACC Case Rep..

[12] Desai A.D., Boursiquot B.C., Moore C.J., Gopinathannair R., Waase M.P., Rubin G.A., Wan E.Y. (2022). Autonomic Dysfunction Post–Acute COVID-19 Infection. Heart Case Rep..

[13] Gyöngyösi M., Alcaide P., Asselbergs F.W., Brundel B.J.J.M., Camici G.G., Martins P.d.C., Ferdinandy P., Fontana M., Girao H., Gnecchi M. (2023). Long COVID and the Cardiovascular System-Elucidating Causes and Cellular Mechanisms in Order to Develop Targeted Diagnostic and Therapeutic Strategies: A Joint Scientific Statement of the ESC Working Groups on Cellular Biology of the Heart and Myocardial and Pericardial Diseases. Cardiovasc. Res..

[14] Cole A.P., Perry D., Hobbs J.R. (1977). Abnormalities of Immunoglobulins in Infants with Congenital Heart Disease. Acta Paediatr..

[15] Singampalli K.L., Jui E., Shani K., Ning Y., Connell J.P., Birla R.K., Bollyky P.L., Caldarone C.A., Keswani S.G., Grande-Allen K.J. (2021). Congenital Heart Disease: An Immunological Perspective. Front. Cardiovasc. Med..

[16] Han E., Müller-Zlabinger K., Hasimbegovic E., Poschenreithner L., Kastner N., Maleiner B., Hamzaraj K., Spannbauer A., Riesenhuber M., Vavrikova A. (2025). Circulating Autoantibodies Against Vasoactive Biomarkers Related to Orthostatic Intolerance in Long COVID Patients Compared to No-Long-COVID Populations: A Case-Control Study. Biomolecules.

[17] Aranyó J., Bazan V., Lladós G., Dominguez M.J., Bisbal F., Massanella M., Sarrias A., Adeliño R., Riverola A., Paredes R. (2022). Inappropriate Sinus Tachycardia in Post-COVID-19 Syndrome. Sci. Rep..

[18] Dmytrenko O., Lavine K.J. (2022). Cardiovascular Tropism and Sequelae of SARS-CoV-2 Infection. Viruses.

[19] Tsai E.J., Čiháková D., Tucker N.R. (2023). Cell-Specific Mechanisms in the Heart of COVID-19 Patients. Circ. Res..

[20] Navaratnarajah C.K., Pease D.R., Halfmann P.J., Taye B., Barkhymer A., Howell K.G., Charlesworth J.E., Christensen T.A., Kawaoka Y., Cattaneo R. (2021). Highly Efficient SARS-CoV-2 Infection of Human Cardiomyocytes: Spike Protein-Mediated Cell Fusion and Its Inhibition. J. Virol..

[21] Sakai Y., Imai S., Sato Y., Yagi H., Kushiro T. (2006). Clinical and Electrophysiological Characteristics of Binodal Disease. Circ. J..

[22] Phillips C.M., Smyth J.W. (2025). Viral Infection and Connexin Dysfunction in the Heart. Curr. Cardiol. Rep..

[23] Pierucci N., Mariani M.V., Iannetti G., Maffei L., Coluccio A., Laviola D., Palombi M., Trivigno S., Spadafora L., Chourda E. (2025). Atrial Cardiomyopathy: New Pathophysiological and Clinical Aspects. Minerva Cardiol. Angiol..

[24] Goette A., Corradi D., Dobrev D., Aguinaga L., Cabrera J.-A., Chugh S.S., De Groot J.R., Soulat-Dufour L., Fenelon G., Hatem S.N. (2024). Atrial Cardiomyopathy Revisited—Evolution of a Concept: A Clinical Consensus Statement of the European Heart Rhythm Association (EHRA) of the ESC, the Heart Rhythm Society (HRS), the Asian Pacific Heart Rhythm Society (APHRS), and the Latin American Heart Rhythm Society (LAHRS). Europace.

